# Physical Properties and Effect in a Battery of Safety Pharmacology Models for Three Structurally Distinct Enteric Polymers Employed as Spray-Dried Dispersion Carriers

**DOI:** 10.3389/fphar.2016.00368

**Published:** 2016-10-13

**Authors:** Ryan M. Fryer, Mita Patel, Xiaomei Zhang, Katja S. Baum-Kroker, Akalushi Muthukumarana, Brian Linehan, Yin-Chao Tseng

**Affiliations:** ^1^Cardiometabolic Diseases Research, Boehringer Ingelheim Pharmaceuticals Inc.Ridgefield, CT, USA; ^2^Small Molecule Discovery Research, Boehringer Ingelheim Pharmaceuticals Inc.Ridgefield, CT, USA; ^3^Drug Discovery Support, Boehringer Ingelheim Pharmaceuticals Inc.Biberach, Germany

**Keywords:** amorphous solid dispersion, spray-dried dispersion, safety pharmacology, drug discovery

## Abstract

Establishing a wide therapeutic index (TI) for pre-clinical safety is important during lead optimization (LO) in research, prior to clinical development, although is often limited by a molecules physiochemical characteristics. Recent advances in the application of the innovative vibrating mesh spray-drying technology to prepare amorphous solid dispersions may offer an opportunity to achieve high plasma concentrations of poorly soluble NCEs to enable testing and establishment of a wide TI in safety pharmacology studies. While some of the amorphous solid dispersion carriers are generally recognized as safe for clinical use, whether they are sufficiently benign to enable *in vivo* pharmacology studies has not been sufficiently demonstrated. Thus, the physical properties, and effect in a battery of *in vivo* safety pharmacology models, were assessed in three classes of polymers employed as spray-dried dispersion carriers. The polymers (HPMC-AS, Eudragit, PVAP) displayed low affinity with acetone/methanol, suitable for solvent-based spray drying. The water sorption of the polymers was moderate, and the degree of hysteresis of HPMC-AS was smaller than Eudragit and PVAP indicating the intermolecular interaction of water-cellulose molecules is weaker than water-acrylate or water-polyvinyl molecules. The polymer particles were well-suspended without aggregation with a mean particle size less than 3 μm in an aqueous vehicle. When tested in conscious Wistar Han rats in safety pharmacology models (*n* = 6–8/dose/polymer) investigating effects on CNS, gastrointestinal, and cardiovascular function, no liabilities were identified at any dose tested (30–300 mg/kg PO, suspension). In brief, the polymers had no effect in a modified Irwin test that included observational and evoked endpoints related to stereotypies, excitation, sedation, pain/anesthesia, autonomic balance, reflexes, and others. No effect of the polymers on gastric emptying or intestinal transit was observed when measured using a barium sulfate tracer material. Finally, in telemetry-instrumented rats the polymers had no effect on acute or 24-h mean blood pressure and heart rate values at doses up to 300 mg/kg. Thus, the properties of the three enteric polymers are appropriate as spray-dried dispersion carriers and were benign in a battery of safety pharmacology studies, demonstrating their applicability to enable *in vivo* safety pharmacology profiling of poorly soluble molecules during LO.

## Introduction

Safety pharmacology studies in drug discovery are often performed during compound selection in lead optimization, prior to entry into long-term dose-escalation studies (Hamdam et al., 2013). Moreover, to protect the Phase I volunteer, *in vivo* studies are mandated by regulatory authorities in key organ systems, including testing in models to investigate effects on CNS, respiratory, and cardiovascular function (USDHHS, 2001). Effects on other organ systems (e.g., gastrointestinal and renal function) are also commonly assessed as part of a comprehensive battery of *in vivo* safety profiling (Al-Saffar et al., 2015; Benjamin et al., 2015).

While establishing a wide therapeutic index (TI) in these models is important prior to advancement of new chemical entities (NCEs) into clinical development, molecules are often encountered with poor aqueous solubility and dissolution characteristics, and consequently, a poor pharmacokinetic profile that limits the ability to achieve high plasma levels after oral administration. Thus, to establish a wide TI for poorly soluble molecules the use of enabling formulations is essential (Li and Zhao, 2007) and whereby the formulation does not interfere with the primary safety endpoints being measured. While some excipients are recognized as safe for clinical use, animal species may respond differently as recently discussed by Turner et al. (2011) who note that some excipients believed to be safe in man, non-human primates, and dogs are not well tolerated in rodents, and vice versa. Moreover, excipient tolerance can be impacted by the fasted/fed state of the animal who may become less tolerant of excipients as their plane of nutrition decreases (Li and Zhao, 2007).

While an enteric polymer, such as those tested in the present study, would not be absorbed into the blood, it is well established that within the digestive system, GI reflexes can be modulated through both systemic as well as complex local and regional effects processed entirely within the digestive system itself (e.g., to control secretion and local motility via gastro-colic, entero-gastric, and colono-ilial reflexes) ultimately affecting GI function (Furness et al., 2014; Al-Saffar et al., 2015). The importance of proper formulation excipient characterization has also been noted specifically in support of small-animal telemetry profiling for cardiovascular safety (Guth, 2007) to enable detection of the low-magnitude yet physiologically-relevant effects, and where volume, dose rate, and physicochemical characteristics of the excipient are critical factors impacting hemodynamic responses (Authier et al., 2015). The same principles apply to CNS functional assessments where prior formulation evaluation is essential when comparing subjective scores as commonly employed in a Functional Observational Battery or Irwin profile (Fonck et al., 2015).

Thus, a proper balance between selecting a formulation to achieve high drug levels *vs*. biocompatibility and *in vivo* tolerability must be developed to minimizing confounding effects of the formulation excipients (Li and Zhao, 2007). The latter is highlighted in the observation that some enabling solution formulations are not suitable for *in vivo* evaluation due to the adverse effect profile of vehicles composed of organic solvents, surfactants, lipids, or complexing agents (Pestel et al., 2006). The amorphous solid dispersion approach is considered one of the most promising strategies to address bioavailability issues that are mediated by solubility and dissolution-limited absorption (Vasconcelos et al., 2007; Padden et al., 2010; Newman et al., 2012). In an amorphous solid dispersion system, the carrier plays a critical role because the carrier is a determinant of physical stability, dissolution, and supersaturation of the amorphous drug. The development of amorphous solid dispersions using pre-selected carriers that can enhance plasma exposure and are also suitable for *in vivo* use is of significant value in pharmacology studies where testing at pharmacologic or supratherapeutic doses is limited by less-than-ideal physiochemical characteristics of the molecule. Moreover, added value is evident for those pharmacology sub-specialties whereby administration at large, often supratherapeutic, doses is necessary to achieve a wide TI as dictated in pre-clinical safety pharmacology studies and longer-term toxicological profiling.

While amorphous solid dispersions have been increasingly used to enhance bioavailability of poorly soluble molecules at later stages of the drug development process, this approach has had limited utility during lead optimization. This is primarily because preparing reliable amorphous solid dispersions at the milligram scale is technically challenging, requiring considerable development time and drug substance. However, in previous studies we have demonstrated that utilization of the B-90 spray drier (B-90) using vibrating mesh technology is an effective way to prepare amorphous spray-dried dispersions in the discovery research stages (Gu et al., 2015; Tseng, 2015).

In the present study, we evaluated three enteric polymers from three distinct classes and with different structures that are commonly used as amorphous solid dispersion carriers including cellulose-based hydroxypropylmethylcellulose acetate succinate-LF (HPMC-AS), acrylate-based EUDRAGIT L100-55 (Eudragit), and polyvinyl-based polyvinyl acetate phthalate (PVAP). These enteric polymers were selected for this studies because they dissolve at pH > 5.5 and are not soluble in acidic gastric fluid and water which can prevent the pre-mature release of the incorporated drug molecule during the preparation before dosing.

Although these polymers are generally recognized as safe for clinical use, to the best of our knowledge very few studies have been performed to demonstrate that these polymers are sufficiently benign in pre-clinical models to enable *in vivo* safety pharmacology studies where small changes in organ function and physiology are assessed during lead optimization. The current studies focused on the comparison of the basic physicochemical properties of these three enteric polymers as spray-dried dispersion carriers and their tolerability in safety pharmacology models investigating effects on CNS, gastrointestinal, and cardiovascular function.

## Materials and methods

### Materials

HPMC-AS was purchased from Shin-Etsu Chemical Co. Ltd. (Tokyo, Japan). EUDRAGIT L 100-55 and PVAP were provided by Degussa GmbH (Linden, NJ) and by Colrcon, Inc. (West Point, PA), respectively. All other chemical reagents used in this study were of ACS-grade purity.

### Preparation of spray-dried powders

Spray-dried powders were prepared by means of spray drying using B-90 (BÜCHI Labortechnik AG, Flawil, Switzerland). HPMC-AS was dissolved in acetone, Eudragit in a mixture of acetone and methanol (8/2), and PVAP in acetone and methanol (9/1) to produce 1% (w/v) solution solutions for spray drying. After centrifuging each solution at 3000 rpm for 15 min, the supernatants were spray dried. For the spray-drying process, the pump speed, air flow rate, and inlet temperature were set at 2, 120 L/min, and 75°C, respectively, and a 7-mm mesh nozzle cap was used. Dried powders were collected, and the collected powders were further dried in a vacuum oven at 40°C for 3 days.

### Thermogravimetric analysis (TGA)

The prepared spray-dried powders were analyzed using a TGA Q500 from TA instruments (New Castle, DE) to determine the moisture and volatile contents. The samples were heated at 10°C/min from 25 to 200°C in an inert environment maintained by dry N_2_ purge at 100 mL/min. The percentage weight loss integrated up to 110°C was accounted for the moisture and solvent loss. Data analysis utilized Universal Analysis 2000 thermal analysis software by TA Instruments.

### Dynamic vapor sorption (DVS)

Dynamic gravimetric vapor sorption of spray-dried polymers was done by DVS (Surface measurement systems, Allentown, PA) using water as the solvent. Approximately 5–20 mg of sample was placed into the instrument microbalance where it was dried at 25°C until constant weight was recorded. The dried sample was then exposed to differing humidity's up to 90% RH at 25°C. Mass change was recorded at different humidity's. The equilibrium criterion was 0.0015% mass change. The Adsorption/desorption isotherm curve was plotted using the DVS software.

### Light microscopy and particle size measurement

Spray-dried powders were suspended in an aqueous solution of 0.5% methylcellulose (MC) and 0.015% Tween 80, or in the McIlvaine buffer at pH6. The suspension or solution was sandwiched between a glass slide and a coverslip and visualized under a light microscope of Nikon Eclipse Ti (Nikon, Tokyo, Japan). Microscope images were taken in the PLM mode from an attached Nikon DS-Ri2 digital camera (Nikon, Tokyo, Japan). Particle size measurements were performed on the captured images using NIS-Element Imaging software. Average particle size was expressed as the area mean diameter, and D50 and D90 are the particle diameters determined respectively at the 50th and 90th percentiles of undersized particles.

### *In vivo* safety pharmacology studies

All *in vivo* experiments and procedures were performed under protocols approved by the Boehringer Ingelheim Institutional Animal Care and Use Committee. Procedures involving animals and their care were either conducted in conformity with the United States Animal Welfare Act or the European Union guidelines (EEC Council Directive 86/609). The *in vivo* experiments performed in Germany (gastrointestinal function) were approved by the Ethical Committee of the responsible regional council (Tübingen).

The three spray-dried polymers (HPMC-AS, Eudragit and PVAP) were tested in suspension at 30, 100, and 300 mg/kg p.o. (10 mL/kg) in conscious male Wistar Han rats (Charles River, Kingston NY, US; or Sulzfeld, Germany). CNS functional studies were performed at *n* = 6/dose of each polymer. GI functional studies were performed at *n* = 7–8/dose of each polymer. Cardiovascular studies were performed at *n* = 8/dose of each polymer. Results were compared to a 0.5% methylcellulose/0.015% Tween 80 control vehicle in dH2O and detailed statistical methods are provided below.

#### CNS function

The systematic observation of animal behavior and psychological state, originally described by Irwin (1968) is a widely used method to determine the side effect liability potential of novel agents on CNS function. In a modified Irwin study (*n* = 6/dose) that assessed effects on CNS function at 1, 2, and 4 h post-treatment, rats (weight range = 165–275 g) were placed in observation cylinders for evaluation followed by brief handling and manipulation testing. The number of animals used in the study is based on prior experience with the model where *n* = 6 has been sufficient to demonstrate effects of positive control agents including d-amphetamine, diazepam, and others *(results not shown)*. The modified Irwin study assessed six broad categories of CNS function including stereotypy, excitation, sedation, pain/anesthesia, autonomic balance, reflexes, and other non-categorized effects; specific parameters that were assessed included motor activity, ataxia, paralysis, tremor, convulsions, respiration, salivation, skin color, feces, death, righting reflex, visual orientation, grip reflex, muscle tone, body temperature, and tail pinch. Effects, when noted, were qualitatively characterized as a mild, moderate, or marked response. Relative to vehicle controls an observation was considered “infrequent” if a response was observed in 50% or fewer animals and was deemed mild in magnitude. An effect was described as “limited observations” if observed in 50% or fewer animals at a mild or moderate magnitude. An observation was considered “frequently observed” of observed in greater than half of the animals of any magnitude (mild, moderate, or marked severity). Results were summarized for each vehicle across the six broad categories in **Table 2**.

#### GI function

Indices of gastrointestinal (GI) function (gastric emptying and GI transit) were assessed at 2-h post-dose (*n* = 7–8/dose). The number of animals used in the study is based on prior experience with the model testing including the evaluation of positive control agents including codeine, carbachol, and others *(results not shown)*. Thirty minutes prior to assessment rats (7 weeks of age, 130–160 g) were administered a barium sulfate test meal suspension, an opaque substance easily visualized in the GI tract; barium sulfate was formulated as 10 g BaSO_4_ in 10 mL salt-free water and administered at 20 mL/kg body weight by oral gavage. At 2-h post-dose, and after 30-min of barium sulfate transit time, rats were euthanized and the intestine/stomach removed (stomach to cecum). The extent of barium sulfate GI transit was expressed as a percent of the total intestine length. Gastric emptying was reported as the difference in full and empty dry weight of the stomach. The percent effect on both transit and gastric emptying was calculated relative to vehicle (mean ± SD).

#### Hemodynamics

Male Wistar Han rats (*n* = 8/dose) were instrumented with telemetry transmitters (DSI, St. Paul, MN) as previously described (Fryer et al., 2012a) to continuously record hemodynamic parameters while conscious and freely moving after oral administration. Group size was based on prior experience with the model and as previously described (Fryer et al., 2012b). All animals (28–30 weeks of age, 357–521 g) were single housed and acclimated to metabolic cages for 3 days. Blood pressure, heart rate and bodyweight were collected during a 24-h baseline period and animals were randomized. Effects on mean arterial blood pressure and heart rate were assessed reported as 10 min mean values for 24 h of baseline and 24-h post-administration.

#### Statistical analysis

Statistical analyses were performed for studies investigating effects on GI and cardiovascular function; since only observational endpoints were assessed in the modified Irwin profile no statistical analysis was performed for those studies. In GI functional studies statistical significance was determined separately for gastric emptying and GI transit endpoints and separately for each polymer. For each polymer, values were assessed at all doses tested using a one-way ANOVA and Dunnett's multiple comparison to the vehicle control group; *p* < 0.05 was considered statistically significant (GraphPad Prism 6.0). In studies performed in telemetry rats investigating effects on hemodynamics, two separate statistical analyses were performed. Baseline values (mean arterial blood pressure and heart rate) were defined as the average of all values collected during the 24-h period prior to treatment; values were compared using a one-way ANOVA and Dunnett's multiple comparison to the vehicle control group; *p* < 0.05 was considered statistically significant (GraphPad Prism 6.0). For analysis of individual timepoints during the 48-h period, a two-way ANOVA with Dunnett's multiple comparison test *vs*. the Control Vehicle group (GraphPad Prism 6.0, *p* < 0.05) was performed based on change from baseline in the hemodynamic values; statistically significant values are denoted within the figures using asterisks in the corresponding group color.

## Results and discussion

**Figure 1:**
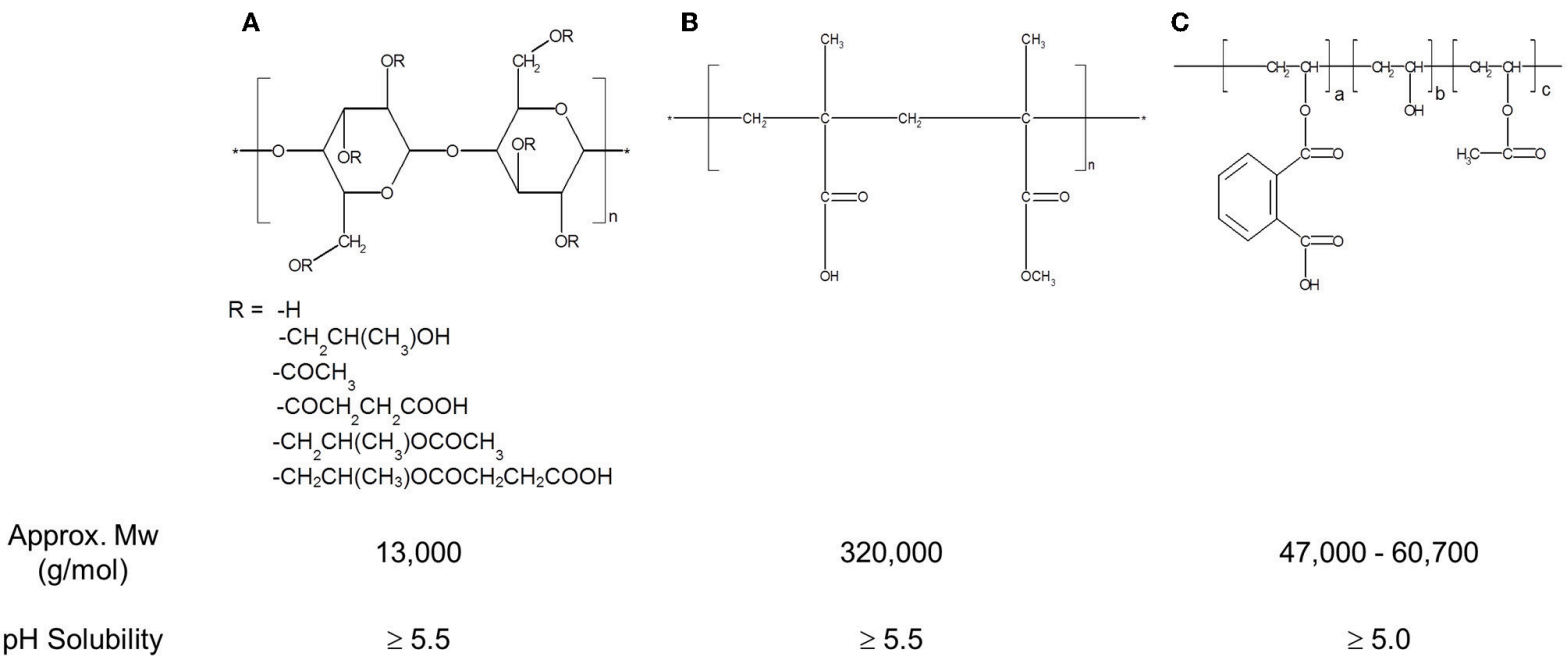
**Structure, MW, and pH-solubility threshold of Hydroxypropylmethylcellulose acetate succinate (HPMC-AS) (A), Methacrylic Acid-Ethyl Acrylate Copolymer (1:1) (Eudragit® L 100-55) (B), and Polyvinyl acetate phthalate (PVAP) (C)**.

**Figure 2:**
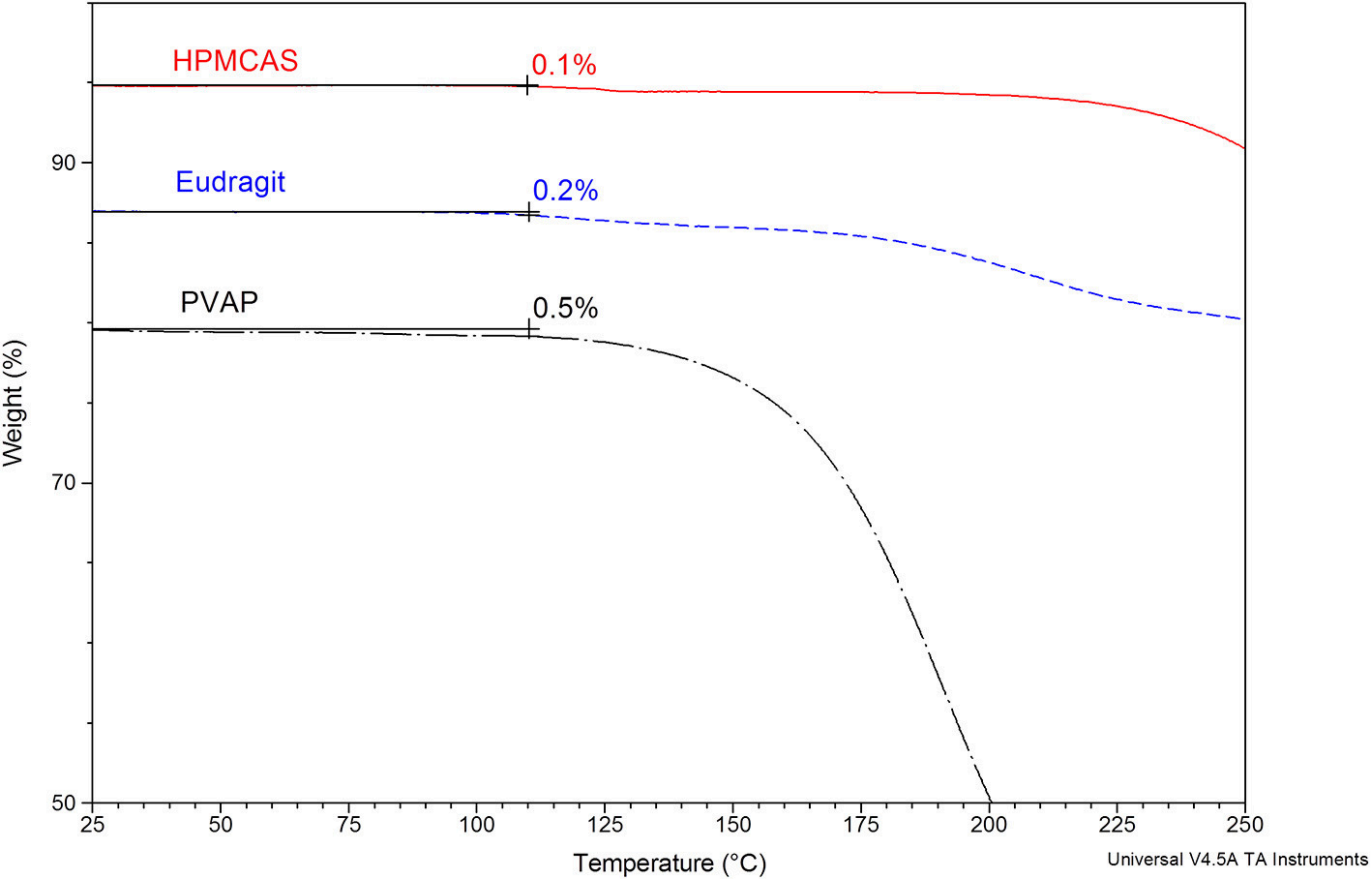
**Thermogravimetric analysis (TGA) of spray-dried HPMC-AS, Eudragit, and PVAP**. The percentage weight loss integrated up to 110°C is accounted for the moisture and solvent loss.

The structures, average molecular weight, and pH-solubility threshold of the three selected polymers are shown in Figure 1. They are ionic polymers, and their pH-solubility thresholds are 5.5, 5.5, and 5.0 for HPMC-AS, Eudragit, and PVAP, respectively. This means that the polymers are not soluble in acidic gastric fluid, but swell and dissolve rapidly at a pH > 5.5. All three polymers as reported in the literature have good solubility in acetone, methanol, or their combination (Rowe et al., 2006; Sullivan, 2008; Evonik Nutrition and Care Gmb, 2015). A key prerequisite for preparing amorphous solid dispersions with the solvent-based spray drying technology is to dissolve a compound and a polymer carrier in a common volatile solvent (typically acetone or methanol), followed by spray-drying. During the spray-drying process, the solvent rapidly evaporates from the droplets to rapidly solidify the polymer and drug mixture, trapping the drug as an amorphous molecular dispersion, but the solvents may also get entrapped in the spray-dried powders (Sollohub and Cal, 2010). In addition to safety concerns, the residual solvent, even at low levels, can cause recrystallization of the amorphous compound in solid dispersions, potentially having a detrimental effect on the stability of the dispersed amorphous compound (Andronis et al., 1997). Therefore, it is important that the amorphous solid dispersion carriers have a minimal affinity with the solvents. The amount of solvent trapped in the spray-dried powders was assessed with TGA for a total volatile content. As shown in Figure 2, there was no entrapped acetone in HPMC-AS, and a very small amount of acetone/methanol was entrapped in Eudragit and PVAP, suggesting a low affinity between the three polymers and the solvents, and HPMC-AS has the lowest affinity.

**Figure 3:**
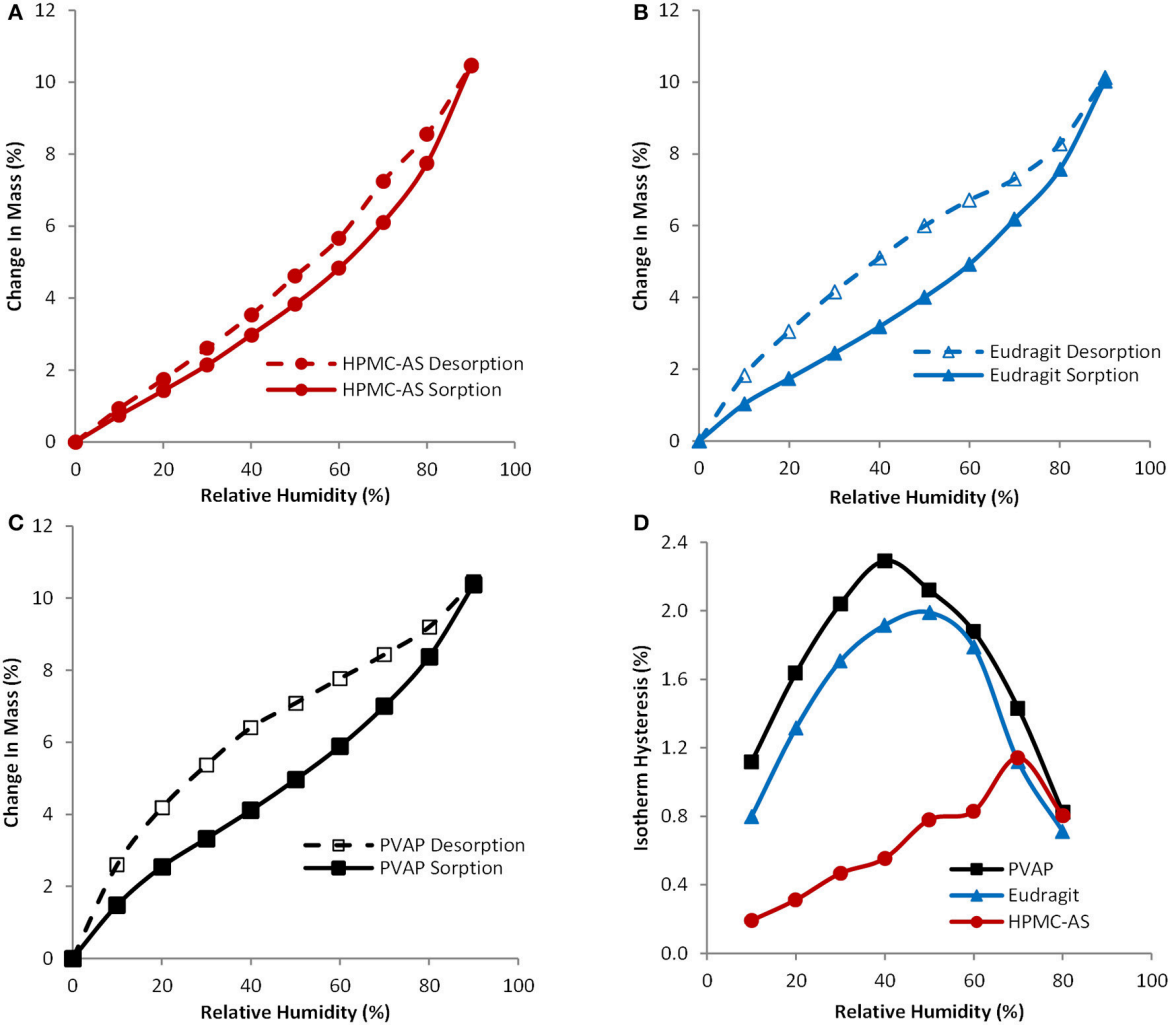
**Sorption and desorption isotherms for spray-dried HPMC-AS (A), Eudragit (B), PVAP (C), and isotherm hysteresis (D) of spray-dried HPMC-AS (●), Eudragit (▲), and PVAP (■)**.

Hygroscopicity of amorphous solid dispersion carriers is recognized as a critical factor in determining the physical stability or crystallization tendency of amorphous compounds, because absorbed water can cause recrystallization of dispersed amorphous compounds. A water vapor sorption isotherm provides information about the affinity between amorphous solid dispersion carriers and water. Figures 3A–C exhibit water vapor sorption and desorption isotherms for HPMC-AS, Eudragit, and PVAP, respectively, and Figure 3D displays hysteresis over the humidity range of 0–90% RH. As shown in Figures 3A–C, these three amorphous polymers, like any other amorphous materials, absorbed water when exposed to humid air. The degree and pattern of water uptake was practically the same for all polymers. The water uptake was slow and continuous through the entire range of humidity, and the observed changes in mass were 10.5, 10.0, and 10.4% for HPMC-AS, Eudragit, and PVAP, respectively, at 90% RH. Other researchers reported that enteric polymers are less hygroscopic than water-soluble polymers such as povidone or copovidone, thereby imparting better stability to the amorphous form (Marsac et al., 2008; Rumondor et al., 2009). The displayed water vapor sorption isotherm is the total sorption contributed by both surface adsorption and bulk absorption (Crowley and Zografi, 2002). The moisture taken by the bulk absorption into the structure of amorphous solid dispersions may induce phase separation of the drug-polymer system and subsequent crystallization of amorphous drugs (Konno and Taylor, 2008; Marsac et al., 2010). As shown in Figures 3A–C, all three polymers had certain degree of hysteresis between the sorption and desorption isotherm, which is mainly induced by the bulk absorption, and water is absorbed more quickly than being released (Crowley and Zografi, 2002). The degree of hysteresis for HPMC-AS is smaller than that of Eugragit and PVAP (Figure 3D), indicating the intermolecular interaction of water-cellulose molecules is weaker than the Eudragit and PVAP. Chokshi et al. (2008) have shown that polymers with ionic groups, such as HPMC-AS, Eudragit or PVAP etc., can help stabilize amorphous systems by not only providing an opportunity for ionic interactions but also by providing low water activity as they are insoluble in water. Rumondor et al. (Rumondor and Taylor, 2010) has also reported that the extent of water sorption depends on the hygroscopicity of both polymer and drug (McConnell et al., 2008). Thus, an amorphous solid dispersion system with a lower hygroscopicity may lead to a greater resistance against moisture-induced phase separation, and the HPMC-AS system containing the least bulk absorption is least susceptible to phase separation.

**Figure 4:**
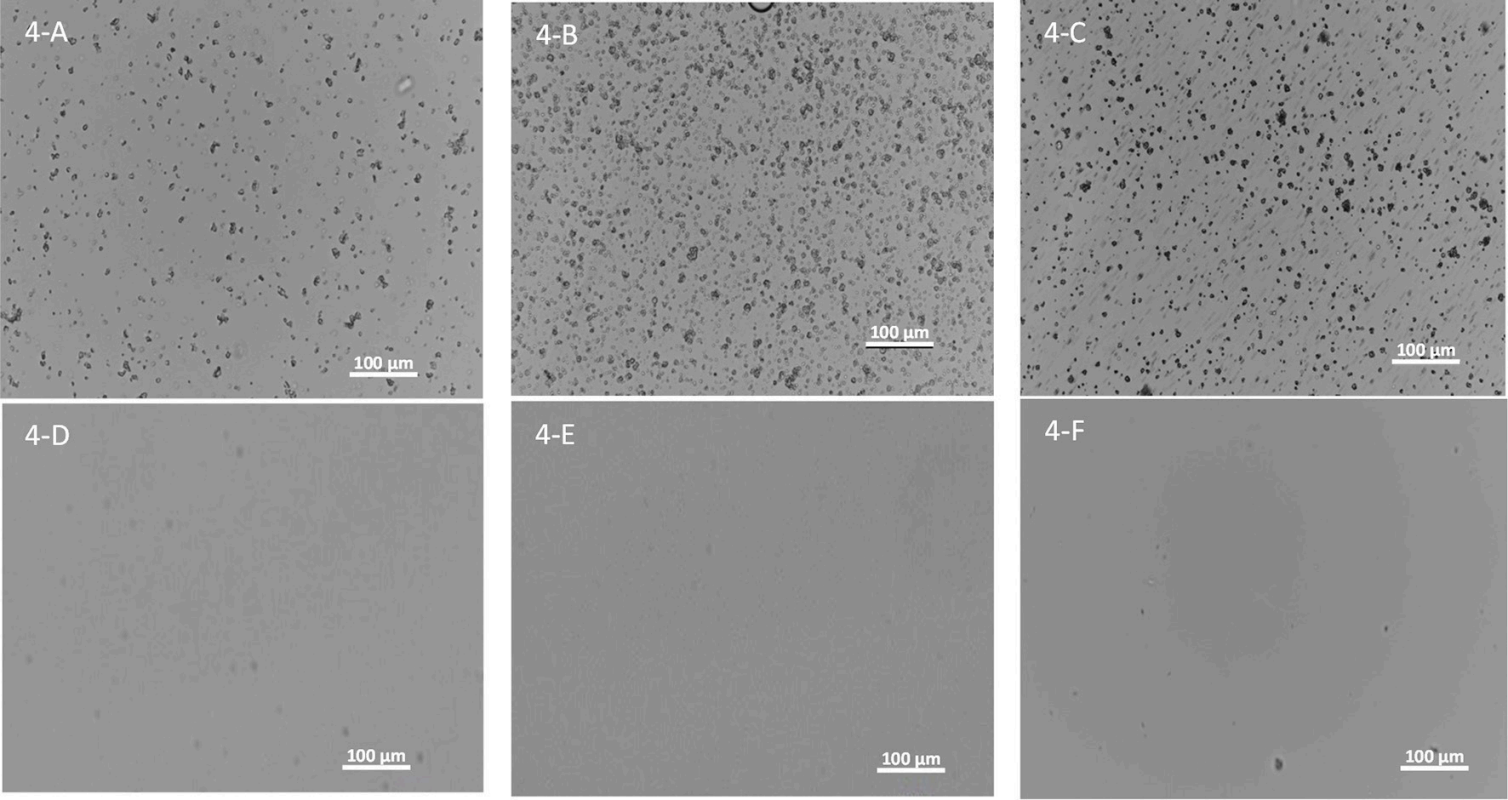
**Images of spray-dried powders of HPMC-AS (A), Eudragit (B), and PVAP (C) dispersed in the aqueous vehicle (0.5% MC and 0.015% Tween) up to 4 h and HPMC-AS (D), Eudragit (E), and PVAP (F) in a pH6 buffer**.

One of the biggest challenges to dose amorphous solid dispersions in early pre-clinical studies, especially for rodents, is that amorphous solid dispersions need to be suspended in aqueous dosing vehicles during the dosing preparation period before dosing. Therefore, it is critical that the amorphous solid dispersion carriers can be homogeneously suspended in the aqueous dosing vehicle without dissolving to prevent pre-maturely releasing the drug and to avoid crystallization of incorporated amorphous compounds at least 2–4 h for dosing preparation. On the other hand, the carriers should dissolve in the small intestine to release the drug for absorption. Particle size of the suspended ASD carriers in the dosing vehicle is also critical since it influences the dissolution. Hence particle size of spray-dried powders of the three polymers dispersed in the aqueous vehicle (0.5% MC and 0.015% Tween) up to 4 h are given in Table 1. As shown in Figure 4, the three spray-dried polymers formed homogenously suspended particles without aggregations in the aqueous vehicle used (0.5% MC and 0.015% Tween) up to 4 h, indicating most of the incorporated compounds can probably be protected by the carriers from pre-maturely releasing in the dosing vehicle. Table 1 shows that the mean diameters are found to be 2.7 ± 2.9, 0.8 ± 0.5, and 0.5 ± 0.3 μm for HPMC-AS, Eudragit, and PVAP, respectively. The 90% of particles are below 5.0, 2.0, and 0.9 μm for HPMC-AS, Eudragit, and PVAP, respectively. As illustrated in Figure 4, they became clear solution at pH6 (above the solubility threshold), which is the average intestinal pH of mice and rats (McConnell et al., 2008), indicating that the incorporated compounds can be released in the upper GI of rodents.

**Table 1:** **Particle size of spray-dried powders of HPMC-AS, Eudragit, and PVAP dispersed in the aqueous vehicle (0.5% MC and 0.015% Tween); ***n*** = 3, 1000–20,000 particles per sample**.

	**Particle size (μm)**			
	**Mean**	**Range**	**D50**	**D90**
HPMC-AS	2.7 ± 2.9	0.15–19	2.0	5.0
Eudragit	0.8 ± 3.8	0.5–11	0.8	2.0
PVAP	0.5 ± 2.3	0.2–4	0.4	0.9

The polymers tested in the present study are generally recognized as safe in humans, and listed in the FDA's Inactive Ingredient Database (IID). However, as referenced earlier, while some excipients are recognized as safe for clinical use, animal species may respond differently (Pestel et al., 2006; Li and Zhao, 2007; Turner et al., 2011). Previously, PVAP was been evaluated in oral toxicity studies in rats (Schoneker et al., 2003) and subchronic dietary studies where no toxicologically meaningful changes were noted (DeMerlis et al., 2014). Additionally, Hoshi et al. (1985a) previously evaluated the toxicological effects of HPMC-AS on organ function and the material was found generally without effect although the studies did not include an Irwin, cardiovascular, or GI transit assessment in rats. And, while a separate study from the same authors indicate that behavior was not affected in rats after administration at 2.5 g/kg, it is unclear what behavioral endpoints were measured in the study beyond mortality (Hoshi et al., 1985b) and thus comparison to results of the present study is not possible.

Eudragit has been tested in rats at doses up to 4800 mg/kg without lethality (Piccinelli and de Feo, 1985). Additionally, the safety and toxicity assessment of neutral methacrylate copolymer has been reviewed in two publications (Eisele et al., 2011, 2013) in support of GRAS evaluation by authors employed at Evonik who reference a number of studies performed in rats at doses up to 2000 mg/kg/d and where it is suggested that oral administration had no adverse toxicity. However, these conclusions cannot be easily substantiated based on several inaccessible references and where support for lack of toxicity is based on unpublished observations [i.e., refer to multiple citations within (Eisele et al., 2011, 2013) by T. Hirikawa, H. Kaneto, K. Kinkel, E. Rossiello, U. Decker, and several studies performed by F. Leuschner]. Thus, while no acute toxicity might be expected, peer-reviewed studies related specifically to the safety of Eudragit, including Eudragit L-100-55, on CNS, GI, and CV organ systems were not readily available. Therefore, the effects of Eudragit and the other polymers were systematically tested in a battery of *in vivo* models in rats commonly used in safety pharmacology assessment.

Prior to first-in-human studies, pre-clinical safety pharmacology assays, tests, and models are positioned to predict the clinical risk profile of NCEs. More importantly, properly conducted safety pharmacology studies are important given the necessity to reduce the number of late-stage failures by way of triaging drug candidates early and allowing for advancement of the best drug candidates based on a balance between efficacy and safety (Morimoto et al., 2015). While most pre-clinical *in vivo* safety pharmacology models employ quantitative and objective measurements of organ function (e.g., blood pressure in telemetry-instrumented rodents), CNS functional profiling is more difficult to quantify as assessments are typically performed with the use of behavioral models that are largely, albeit not exclusively, dependent on multiple subjective endpoints. Indeed, classical methods to evaluate CNS functional effects consist of observing, and scoring, behavioral responses. The Irwin (1968) and the functional observational battery (Moser et al., 1995) are the most commonly used assays in CNS safety assessment that meet ICHS7A guidelines for pre-clinical assessment of NCEs prior to testing in man (USDHHS, 2001). In the present study that used a Modified Irwin model, when the enteric polymers were evaluated at 1, 2, and 4-h post-dose and relative to the control group, none elicited any marked or consistently dose-dependent effects on any of the broad categories of CNS function including stereotypies, excitation, sedation, pain/anesthesia, autonomic balance, and reflexes (Summarized in Table 2). While some qualitative observations were recorded, none appeared related to administration of the enteric polymers and were not qualitatively different than animals that received the control vehicle. Additionally, while diarrhea was observed in the HPMC-AS studies at the two later timepoints (2 and 4-h p.a.), the effect was observed in only a limited number of animals and was not observed in subsequent studies designed to specifically address effects on GI functional endpoints (gastric emptying and gastrointestinal transit time). Thus, results from CNS safety studies using a modified Irwin protocol demonstrate no untoward effect of the three enteric polymers on CNS function when tested at high doses, up to 300 mg/kg, and demonstrate that all three are suitable for testing novel NCEs during pre-clinical safety evaluation. Moreover, these results appear consistent with a separate study investigating the pharmacological effects of HPMC-AS whereby Hoshi et al. (1985a) demonstrated no effect on generalized behavior in beagles and no effect on hexobarbital-induced sleeping time (125–150 mg/kg, p.o.) or motor coordination (250 mg/kg, p.o.) in rats.

**Table 2:** **Summarized observations from modified Irwin test performed at 1, 2, and 4 h post-dose for PVAP, Eudragit, and HPMC-AS at 30, 100, and 300 mg/kg (***n*** = 6/group/dose) based on Irwin Score**.

**Category**	**Vehicle**	**PVAP**	**Eudragit**	**HPMC-AS**
Stereotypy	–	infrequent, non-DD (chewing/grooming)	–	–
Excitation	–	infrequent, non-DD (increased motor activity)	–	–
Sedation	–	infrequent, non-DD (decreased motor activity, grip reflex)	infrequent, non-DD (decreased motor activity)	infrequent, non-DD (decreased motor activity, muscle tone, grip reflex)
Pain	–	–	–	–
Autonomic	–	–	–	limited observations, DD (diarrhea)
Reflex	–	–	–	–
Body Temperature	–	–	–	–
Paralysis/Death	–	–	–	–

*Lack of effect is denoted by a (–) in the table. Effects, when noted for specific endpoints, are listed in the table and categorized as either (1) observed infrequently, (2) observed in a limited number of animals, or (3) were consistently observed. DD indicates dose-dependency, Non-DD indicates an observation was not dose-dependent*.

**Figure 5:**
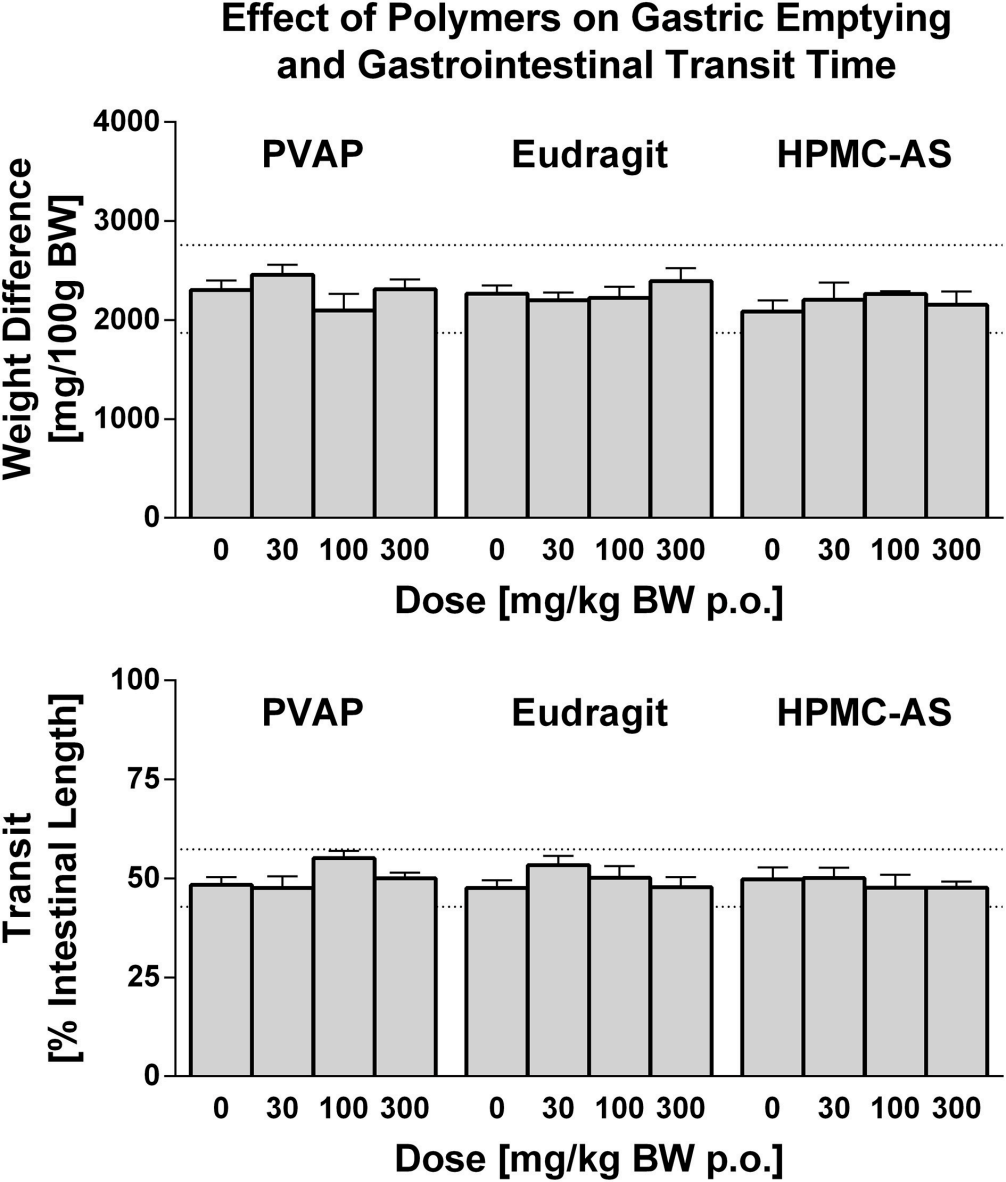
**Effect of PVAP, Eudragit, and HPMC-AS enteric polymers on GI Function in Conscious Rats**. Gastric emptying **(top)** is shown as weight before and after removal of stomach contents (predominately BaSO_4_ test meal) at 3 doses of each polymer (30, 100, or 300 mg/kg; *n* = 7–8/group) in comparison to the control vehicle (MC/Tween) as shown in the 0 mg/kg bar. The extent of BaSO_4_ traverse through the small intestine, indicative of intestinal transit **(bottom)**, is shown as a percent of the total intestine length. The mean range of historical control values for both parameters is shown in dashed lines on the y-axis.

Historically, drug attrition due to GI-related adverse effects has been small although reports of this nature have increased over the last few years and tend to be quite diverse, usually functional in nature, and not limited to a single mechanism or therapeutic area (Al-Saffar et al., 2015). Pre-clinical models have been established to screen NCEs for effects on GI function including gastric absorption, secretion, and emptying, gastrointestinal motility, as well as nausea and emesis liabilities (Al-Saffar et al., 2015). Drug-induced effects on GI function can be assessed as endpoints integrated into toxicology studies (Redfern, 2015) or, as in the present study, in stand-alone safety pharmacology studies. In the present investigation, studies were performed using a barium sulfate test meal to assess effects on gastric emptying and GI transit time. The extent of gastric emptying and GI transit time was highly consistent in animals that received the control vehicle in each of the polymer studies. Moreover, no effects on either gastric emptying or GI transit time were observed for any enteric polymer tested at any dose level up to 300 mg/kg (Figure 5). Consistent with these findings, Hoshi et al. (1985a) demonstrated no effect on contraction of the isolated ileum of guinea pigs at doses up to 250 mg/kg. In rats, the study demonstrated no significant effect of HMPC-AS on BaSO_4_ transit in the small intestine, gastric secretions, and bile excretion. Thus, these results demonstrate that the three enteric polymers tested, HPMC-AS, PVAP, and Eudragit, are benign with regard to direct or indirect effects on gastrointestinal function and are suitable for formulation during pre-clinical safety screening in drug discovery.

**Figure 6:**
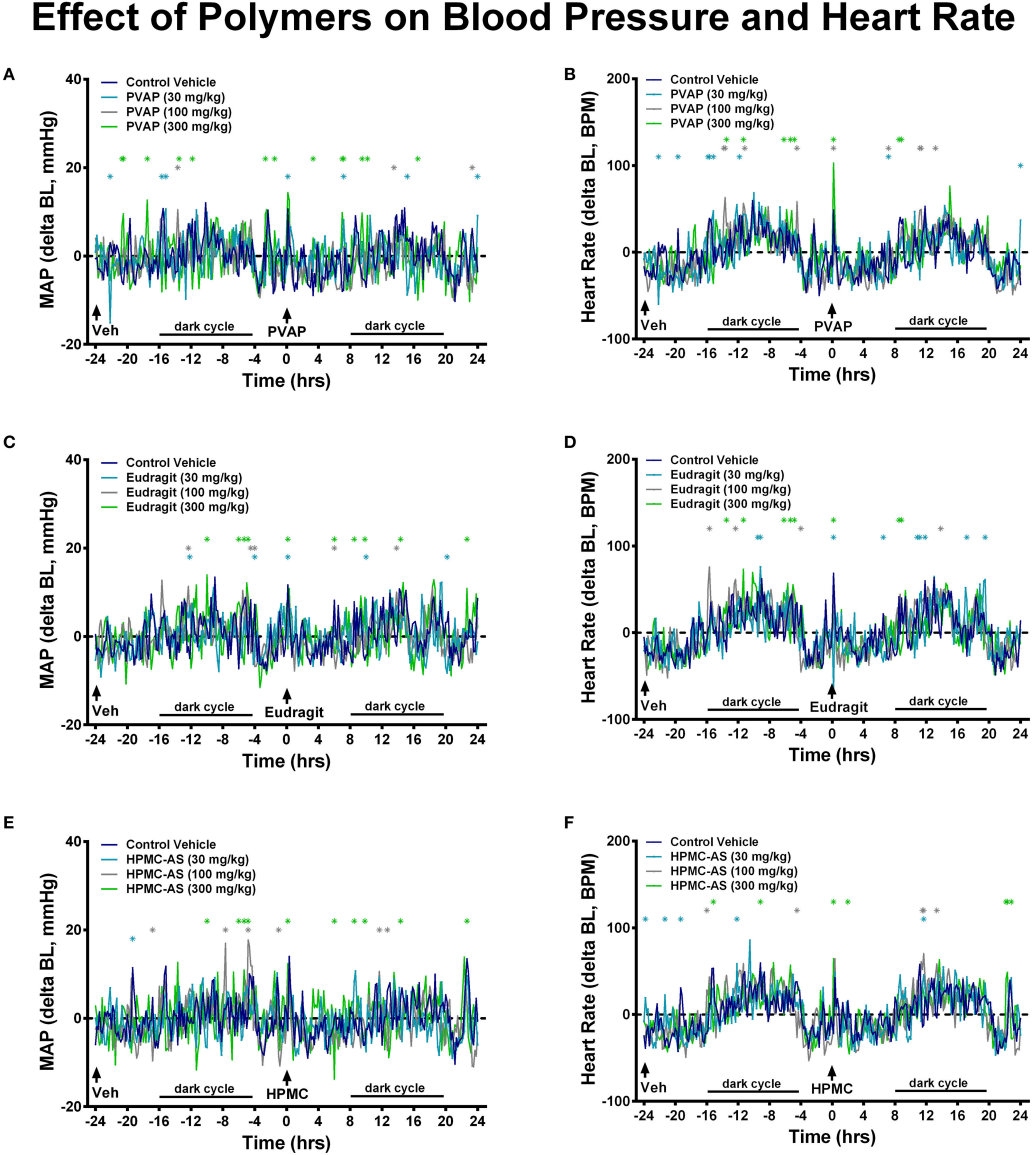
**Effect of PVAP (A,B), Eudragit (C,D), and HPMC-AS (E,F) on cardiovascular function**. Mean values are shown as change from baseline at 10-min intervals during a 48-h continuous recording in telemetry-instrumented conscious rats. The control vehicle was dosed to all animals (*n* = 8/group) at −24 h; at 0 h animals received the control vehicle or polymer at 30, 100, or 300 mg/kg. Values statistically different than the control vehicle group are marked with asterisks; light and dark cycle are also marked that corresponded to diurnal changes in the hemodynamic values.

Hemodynamics, including blood pressure and heart rate, represent important safety pharmacology endpoints with well-established correlation to clinical observations in man (Authier et al., 2015). This has been demonstrated in multiple studies across therapeutic classes and drug mechanisms, and perhaps is most referenced in regard to the off-target effects of the CETP blocker, torcetrapib (Polakowski et al., 2009; Fryer et al., 2012a; Al-Saffar et al., 2015), a molecule that was terminated during late-stage clinical development after demonstration of higher cardiovascular events and mortality in patents and that was associated with small magnitude changes in systolic pressure (4 mmHg) both clinically and pre-clinically (as reviewed in Authier et al., 2015). Cardiovascular safety pharmacology testing can be performed under acute or chronic dosing conditions to probe underlying risk for effects of differential underlying etiology (e.g., acute modulation of blood pressure provoked by changes in vascular tone vs. chronic modulation of blood pressure due to alterations in Na^+^ balance) (Fryer et al., 2012c). Moreover, since cardiovascular liabilities can be elicited via myriad physiological mechanisms (e.g., alterations in autonomic balance, renal hemodynamics, fluid volume, electrolyte concentrations, vascular tone, cardiac conduction) and impacted by any number of receptors, proteins, kinases, and channels, it is difficult to determine *a priori* what physiochemical characteristics may trigger a cardiovascular response. Indeed, a majority of compounds have been estimated to be abandoned during lead optimization due to cardiovascular liabilities and cardiovascular liabilities remain one of the most cited reason for attrition due to safety in clinical development (Laverty et al., 2011; Ferri et al., 2013; Roberts et al., 2014). However, establishing early SAR around off-target cardiovascular effects has proven an effective strategy to mitigate cardiovascular risk during lead discovery (Kym et al., 2006; Lynch et al., 2006; Fryer et al., 2012b). Thus, establishing a wide TI with regard to cardiovascular safety is often considered a principal optimization point prior to advancement of NCEs into clinical development. To the author's knowledge, only HMPC-AS has been investigated in cardiovascular pharmacology studies. While the study employed i.v. administration of the polymer (25 mg/kg) to beagles, no acute effect on blood pressure or the ECG was observed (Hoshi et al., 1985a). In the present investigation, in cardiovascular studies performed in telemetry-instrumented rats, baseline mean arterial blood pressure and heart rate values were consistent between the vehicle control and groups receiving the enteric polymers (Tables 3, 4). Administration of the enteric polymers (PVAP, Eudragit, or HPMC-AS) at doses from 30 to 300 mg/kg had no effect on either acute hemodynamic values (Figure 6) or chronic hemodynamic values represented as a 24-h mean (Tables 3, 4). Moreover, the polymers had no effect on diurnal fluctuations in heart rate due to light/dark cycles (Figure 6). While some statistically-significant differences were detected *vs*. the control vehicle group at discrete timepoints (measured and shown at 10-min intervals over a 48-h continuous recording), no dose-dependency was observed nor were the number of statistically-significant values increased in the post-dose period (0–24 h) vs. the pre-dose baseline period (from −24 to 0 h). Therefore, results of the present study demonstrate that the three enteric polymers, HPMC-AS, PVAP, and Eudragit are benign with regard to acute cardiovascular function and represent a suitable formulation for testing novel NCEs during pre-clinical cardiovascular safety screening.

**Table 3:** **Twenty-four hour mean blood pressure values (mmHg) at baseline and after administration of an aqueous control vehicle or the enteric polymers from 3 to 300 mg/kg (***n*** = 8/group/dose)**.

**Phase**	**Baseline**				**Post-dose**			
**mg/kg**	**Control**	**30**	**100**	**300**	**Control**	**30**	**100**	**300**
PVAP	97.4 ± 1.6	96.5 ± 1.2	95.6 ± 1.2	97.3 ± 2.2	97.0 ± 1.8	95.9 ± 1.1	93.9 ± 1.0	96.7 ± 2.0
Eudragit	96.7 ± 1.6	95.6 ± 1.1	95.4 ± 1.3	96.3 ± 2.0	97.3 ± 1.7	96.1 ± 1.1	95.2 ± 0.9	96.8 ± 1.8
HPMC-AS	96.3 ± 1.5	96.5 ± 1.1	96.7 ± 1.8	95.2 ± 2.3	96.4 ± 1.6	95.8 ± 1.0	95.6 ± 1.6	95.6 ± 2.1

**Table 4:** **Twenty-four hour mean heart rate values (beats/min) at baseline and after administration of an aqueous control vehicle or the enteric polymers from 3 to 300 mg/kg (***n*** = 8/group/dose)**.

**Phase**	**Baseline**				**Post-dose**			
**mg/kg**	**Control**	**30**	**100**	**300**	**Control**	**30**	**100**	**300**
PVAP	345 ± 5	333 ± 5	343 ± 3	340 ± 3	341 ± 5	330 ± 6	340 ± 3	341 ± 3
Eudragit	345 ± 5	332 ± 6	342 ± 3	343 ± 4	344 ± 5	333 ± 7	344 ± 3	343 ± 4
HPMC-AS	344 ± 6	334 ± 6	343 ± 4	343 ± 4	346 ± 5	336 ± 7	345 ± 4	345 ± 4

The importance of excipient interrogation *a priori* has previously been highlighted by work of Pestel et al. (2006) who investigated the effect of common excipients of dosing vehicles in gastrointestinal, renal, and liver functional studies. They note that poorly controlled studies are not uncommon where a vehicle may be selected based only on dissolution-supporting characteristics without the proper consideration for impact on measured functional endpoints. The authors also highlight the need for evidence-based information to enable the rational selection of solubilizing agents suitable for pharmacological testing (Pestel et al., 2006). Moreover, Li and Zhao (2007) have noted that establishing literature precedent or databases relating formulations to safety endpoints may provide valuable support for early formulations in drug discovery where the question of using excipients without causing adverse effects is at the core of early formulation development. In line with these recommendations, the present studies establish that PVAP, HPMC-AS, and Eudragit are acceptable excipients for *in vivo* safety pharmacology profiling.

## Conclusion

Due to very limited bulk drug supplies in the fast-paced drug discovery environment, it is impractical and almost unfeasible to conduct an extensive full screening of polymers to select the most optimal amorphous solid dispersion carriers for pre-clinical safety pharmacology studies where careful consideration and selection of the proper carrier is essential. To reduce development timelines and consumption of drug substance, a more effective alternative approach has been taken to evaluate pre-selected polymers as amorphous solid dispersion carriers. Based on the aforementioned properties and tolerability of specific polymers (HPMC-AS, Eudragit, and PVAP) representing three classes, we demonstrate that all are amenable as carriers for spray-dried dispersions with appropriate physiochemical properties and, importantly, none of the enteric polymers had any effect on CNS, gastrointestinal, or cardiovascular functional parameters when tested at high doses (up to 300 mg/kg) in rats. Results from the present studies demonstrate the utility of these enteric polymers as spray-dried dispersion carriers to enable the formulation of poorly soluble compounds for pre-clinical safety pharmacology evaluation, and to thereby enable an adequate TI for pre-clinical safety during lead optimization in research and candidate selection.

## Author contributions

RF and YT contributed to the overall concept and majority writing of the manuscript. Pharmaceutical characterization studies of the polymers were performed by MP and BL. CNS functional studies were performed by AM, GI functional studies were performed by KB, and cardiovascular functional studies were performed by XZ. All authors authored their respective methods and results sections, and reviewed and edited the final text.

## Funding

This research received no specific grant from any funding agency in the public, commercial, or not-for-profit sectors.

### Conflict of interest statement

The authors declare that the research was conducted in the absence of any commercial or financial relationships that could be construed as a potential conflict of interest.

## References

[B1] Al-Saffar A. Nogueira da Costa A. Delaunois A. Leishman D. J. Marks L. Rosseels M. L.. (2015). Gastrointestinal safety pharmacology in drug discovery and development. Handb. Exp. Pharmacol. 229, 291–321. 10.1007/978-3-662-46943-9_12 26091645

[B2] Andronis V. Yoshioka M. Zografi G. (1997). Effects of sorbed water on the crystallization of indomethacin from the amorphous state. J. Pharm. Sci. 86, 346–351. 10.1021/js9602711 9050804

[B3] Authier S. Pugsley M. K. Curtis M. J. (2015). Haemodynamic assessment in safety pharmacology. Handb. Exp. Pharmacol. 229, 221–241. 10.1007/978-3-662-46943-9_9 26091642

[B4] Benjamin A. Nogueira da Costa A. Delaunois A. Rosseels M. L. Valentin J. P. (2015). Renal safety pharmacology in drug discovery and development. Handb. Exp. Pharmacol. 229, 323–352. 10.1007/978-3-662-46943-9_13 26091646

[B5] Chokshi R. J. Shah N. H. Sandhu H. K. Malick A. W. Zia H. (2008). Stabilization of low glass transition temperature indomethacin formulations: impact of polymer-type and its concentration. J. Pharm. Sci. 97, 2286–2298. 10.1002/jps.21174 17879977

[B6] Crowley K. J. Zografi G. (2002). Water vapor absorption into amorphous hydrophobic drug/poly(vinylpyrrolidone) dispersions. J. Pharm. Sci. 91, 2150–2165. 10.1002/jps.10205 12226842

[B7] DeMerlis C. C. Schoneker D. R. Borzelleca J. F. (2014). Safety of PVAP and PVAP-T including a 90-day dietary toxicity study in rats and genotoxicity tests with polyvinyl acetate phthalate (PVAP). Food Chem. Toxicol. 70, 231–240. 10.1016/j.fct.2014.04.031 24813760

[B8] Eisele J. Haynes G. Kreuzer K. Rosamilia T. (2013). Characterisation and toxicological assessment of Neutral Methacrylate Copolymer for GRAS evaluation. Regul. Toxicol. Pharmacol. 67, 392–408. 10.1016/j.yrtph.2013.08.019 24012708

[B9] Eisele J. Haynes G. Rosamilia T. (2011). Characterisation and toxicological behaviour of basic methacrylate copolymer for GRAS evaluation. Regul. Toxicol. Pharmacol. 61, 32–43. 10.1016/j.yrtph.2011.05.012 21704668

[B10] Evonik Nutrition Care Gmb H. (2015). Eudragit L100-55. Specification and Test Methods. July. Report No.

[B11] Ferri N. Siegl P. Corsini A. Herrmann J. Lerman A. Benghozi R. (2013). Drug attrition during pre-clinical and clinical development: understanding and managing drug-induced cardiotoxicity. Pharmacol. Ther. 138, 470–484. 10.1016/j.pharmthera.2013.03.005 23507039

[B12] Fonck C. Easter A. Pietras M. R. Bialecki R. A. (2015). CNS adverse effects: from functional observation battery/irwin tests to electrophysiology. Handb. Exp. Pharmacol. 229, 83–113. 10.1007/978-3-662-46943-9_4 26091637

[B13] Fryer R. M. Harrison P. C. Muthukumarana A. Nodop Mazurek S. G. Ng K. J. Chen R. R.. (2012a). Strategic integration of *in vivo* cardiovascular models during lead optimization: predictive value of 4 models independent of species, route of administration, and influence of anesthesia. J. Cardiovasc. Pharmacol. 59, 369–376. 10.1097/FJC.0b013e31824485dd 22179024

[B14] Fryer R. M. Muthukumarana A. Chen R. R. Smith J. D. Mazurek S. N. Harrington K. E.. (2012b). Mitigation of off-target adrenergic binding and effects on cardiovascular function in the discovery of novel ribosomal S6 kinase 2 inhibitors. J. Pharmacol. Exp. Ther. 340, 492–500. 10.1124/jpet.111.189365 22128344

[B15] Fryer R. M. Muthukumarana A. Harrison P. C. Nodop Mazurek S. Chen R. R. Harrington K. E.. (2012c). The clinically-tested S1P receptor agonists, FTY720 and BAF312, demonstrate subtype-specific bradycardia (S1P(1)) and hypertension (S1P(3)) in rat. PLoS ONE 7:e52985. 10.1371/journal.pone.0052985 23285242 PMC3532212

[B16] Furness J. B. Callaghan B. P. Rivera L. R. Cho H. (2014). The enteric nervous system and gastrointestinal innervation: integrated local and central control, in Advances in Experimental Medicine and Biology, Vol. 817, eds Lyte M. Cryan J. F. (New York, NY: Springer) 39–71. 10.1007/978-1-4939-0897-4_3 24997029

[B17] Gu B. Linehan B. Tseng Y. C. (2015). Optimization of the Buchi B-90 spray drying process using central composite design for preparation of solid dispersions. Int. J. Pharm. 491, 208–217. 10.1016/j.ijpharm.2015.06.006 26070248

[B18] Guth B. D. (2007). Preclinical cardiovascular risk assessment in modern drug development. Toxicol. Sci. 97, 4–20. 10.1093/toxsci/kfm026 17351262

[B19] Hamdam J. Sethu S. Smith T. Alfirevic A. Alhaidari M. Atkinson J.. (2013). Safety pharmacology–current and emerging concepts. Toxicol. Appl. Pharmacol. 273, 229–241. 10.1016/j.taap.2013.04.039 23732082

[B20] Hoshi N. Ueno K. Yano H. Hirashima K. Kitagawa H. (1985a). General pharmacological studies of hydroxypropylmethylcellulose acetate succinate in experimental animals. J. Toxicol. Sci. 10(Suppl. 2), 129–146. 10.2131/jts.10.SupplementII_129 3841671

[B21] Hoshi N. Yano H. Hirashima K. Kitagawa H. Fukuda Y. (1985b). Toxicological studies of hydroxypropylmethylcellulose acetate succinate–acute toxicity in rats and rabbits, and subchronic and chronic toxicities in rats. J. Toxicol. Sci. 10(Suppl. 2), 147–185. 10.2131/jts.10.SupplementII_147 3841672

[B22] Irwin S. (1968). Comprehensive observational assessment: Ia. A systematic, quantitative procedure for assessing the behavioral and physiologic state of the mouse. Psychopharmacologia 13, 222–257. 10.1007/BF00401402 5679627

[B23] Konno H. Taylor L. S. (2008). Ability of different polymers to inhibit the crystallization of amorphous felodipine in the presence of moisture. Pharm. Res. 25, 969–978. 10.1007/s11095-007-9331-3 17520180

[B24] Kym P. R. Souers A. J. Campbell T. J. Lynch J. K. Judd A. S. Iyengar R.. (2006). Screening for cardiovascular safety: a structure-activity approach for guiding lead selection of melanin concentrating hormone receptor 1 antagonists. J. Med. Chem. 49, 2339–2352. 10.1021/jm0512286 16570930

[B25] Laverty H. Benson C. Cartwright E. Cross M. Garland C. Hammond T.. (2011). How can we improve our understanding of cardiovascular safety liabilities to develop safer medicines? Br. J. Pharmacol. 163, 675–693. 10.1111/j.1476-5381.2011.01255.x 21306581 PMC3111672

[B26] Li P. Zhao L. (2007). Developing early formulations: practice and perspective. Int. J. Pharm. 341, 1–19. 10.1016/j.ijpharm.2007.05.049 17658228

[B27] Lynch J. K. Freeman J. C. Judd A. S. Iyengar R. Mulhern M. Zhao G.. (2006). Optimization of chromone-2-carboxamide melanin concentrating hormone receptor 1 antagonists: assessment of potency, efficacy, and cardiovascular safety. J. Med. Chem. 49, 6569–6584. 10.1021/jm060683e 17064075

[B28] Marsac P. J. Konno H. Rumondor A. C. Taylor L. S. (2008). Recrystallization of nifedipine and felodipine from amorphous molecular level solid dispersions containing poly(vinylpyrrolidone) and sorbed water. Pharm. Res. 25, 647–656. 10.1007/s11095-007-9420-3 17846870

[B29] Marsac P. J. Rumondor A. C. Nivens D. E. Kestur U. S. Stanciu L. Taylor L. S. (2010). Effect of temperature and moisture on the miscibility of amorphous dispersions of felodipine and poly(vinyl pyrrolidone). J. Pharm. Sci. 99, 169–185. 10.1002/jps.21809 19492305

[B30] McConnell E. L. Basit A. W. Murdan S. (2008). Measurements of rat and mouse gastrointestinal pH, fluid and lymphoid tissue, and implications for *in-vivo* experiments. J. Pharm. Pharmacol. 60, 63–70. 10.1211/jpp.60.1.0008 18088506

[B31] Morimoto B. H. Castelloe E. Fox A. W. (2015). Safety pharmacology in drug discovery and development. Handb. Exp. Pharmacol. 229, 65–80. 10.1007/978-3-662-46943-9_3 26091636

[B32] Moser V. C. Cheek B. M. MacPhail R. C. (1995). A multidisciplinary approach to toxicological screening: III. Neurobeh. Toxic. 45, 173–210. 10.1080/15287399509531988 7783252

[B33] Newman A. Knipp G. Zografi G. (2012). Assessing the performance of amorphous solid dispersions. J. Pharm. Sci. 101, 1355–1377. 10.1002/jps.23031 22213468

[B34] Padden B. E. Miller J. M. Robbins T. Zocharski P. D. Prasad L. Spence J. K. (2010). Amorphous solid dispersions as enabling formulations for discovery and early development. Am. Pharm. Rev. 14, 68–70.

[B35] Pestel S. Martin H. J. Maier G. M. Guth B. (2006). Effect of commonly used vehicles on gastrointestinal, renal, and liver function in rats. J. Pharmacol. Toxicol. Methods 54, 200–214. 10.1016/j.vascn.2006.02.006 16567111

[B36] Piccinelli D. de Feo G. (1985). [Acute toxicity of (2,4,6-trimethoxy) phenyl-3 (pyrrolidinyl-1) propyl-ketone chlorhydrate (buflomedil) and of metacrylic copolymers (eudragit) both alone and in association in the mouse and rat]. Boll. Soc. Ital. Biol. Sper. 61, 1579–1585. 3833274

[B37] Polakowski J. S. King A. J. Campbell T. J. Nelson R. A. Preusser L. C. Kempf-Grote A. J.. (2009). Cardiovascular effects of torcetrapib in conscious and pentobarbital-anesthetized dogs. J. Cardiovasc. Pharmacol. 54, 543–551. 10.1097/FJC.0b013e3181bfb158 19770671

[B38] Redfern W. S. (2015). Inclusion of safety pharmacology endpoints in repeat-dose toxicity studies. Handb. Exp. Pharmacol. 229, 353–381. 10.1007/978-3-662-46943-9_14 26091647

[B39] Roberts R. A. Kavanagh S. L. Mellor H. R. Pollard C. E. Robinson S. Platz S. J. (2014). Reducing attrition in drug development: smart loading preclinical safety assessment. Drug Discov. Today 9, 341–347. 10.1016/j.drudis.2013.11.014 24269835

[B40] Rowe R. C. Sheskey P. J. Owen S. C. (eds.). (2006). Polyvinyl acetate phthalate, in Handbook of Pharmaceutical Excipients, 5th Edn (London: Pharmaceutical Press), 589–591.

[B41] Rumondor A. C. Stanford L. A. Taylor L. S. (2009). Effects of polymer type and storage relative humidity on the kinetics of felodipine crystallization from amorphous solid dispersions. Pharm. Res. 26, 2599–2606. 10.1007/s11095-009-9974-3 19806435

[B42] Rumondor A. C. Taylor L. S. (2010). Effect of polymer hygroscopicity on the phase behavior of amorphous solid dispersions in the presence of moisture. Mol. Pharm. 7, 477–490. 10.1021/mp9002283 20039693

[B43] Schoneker D. R. DeMerlis C. C. Borzelleca J. F. (2003). Evaluation of the toxicity of polyvinylacetate phthalate in experimental animals. Food Chem. Toxicol. 41, 405–413. 10.1016/S0278-6915(02)00234-X 12504173

[B44] Sollohub K. Cal K. (2010). Spray drying technique: II. Current applications in pharmaceutical technology. J. Pharm. Sci. 99, 587–597. 10.1002/jps.21963 19862804

[B45] Sullivan L. M. (2008). Repeated measures. Circulation 117, 1238–1243. 10.1161/CIRCULATIONAHA.107.654350 18316500

[B46] Tseng Y. C. (2015). Solving solubility issues with amorphous solid dispersions. Eur. Pharm. Rev. 20, 28–32.

[B47] Turner P. V. Pekow C. Vasbinder M. A. Brabb T. (2011). Administration of substances to laboratory animals: equipment considerations, vehicle selection, and solute preparation. J. Am. Assoc. Lab. Anim. Sci. 50, 614–627. 22330706 PMC3189663

[B48] [USDHHS] United States Department of Health Human Services Food Drug Administration, Center for Drug Evaluation, Research Center for Biologics Evaluation, Research. (2001). Guidance for Industry: S7A Safety Pharmacology Studies for Human Pharmaceuticals. Rockville, MD.

[B49] Vasconcelos T. Sarmento B. Costa P. (2007). Solid dispersions as strategy to improve oral bioavailability of poor water soluble drugs. Drug Discov. Today 12, 1068–1075. 10.1016/j.drudis.2007.09.005 18061887

